# Severe Ulcerative Gastritis Associated With Pembrolizumab in a Patient With Metastatic Lung Adenocarcinoma: A Case Report and Review of Gastric Immune-Related Adverse Events

**DOI:** 10.7759/cureus.101115

**Published:** 2026-01-08

**Authors:** Matheus de Almeida Costa, Lucas Pacheco Vital Calazans, Lívia de Paula Soares, Pedro Paulo Gatto de Oliveira Thomé

**Affiliations:** 1 Gastroenterology and Hepatology, University of São Paulo, São Paulo, BRA; 2 Heart Institute, University of São Paulo, São Paulo, BRA

**Keywords:** endoscopy, gastritis, immune checkpoint inhibitors, immune-related adverse events, lung adenocarcinoma, pembrolizumab, ulcerative gastritis

## Abstract

Immune checkpoint inhibitors (ICIs) have revolutionized oncology but trigger unique immune-related adverse events (irAEs). While intestinal toxicities like enterocolitis are frequent, serious upper gastrointestinal (UGI) involvement, such as isolated severe ulcerative gastritis, remains rare. We report the case of a 72-year-old female with metastatic lung adenocarcinoma who developed Grade 3 ulcerative gastritis after eight months of pembrolizumab therapy. The patient presented with a four-week history of epigastric pain, vomiting, and an 8-kg weight loss, complicated by severe iron-deficiency anemia. Endoscopy revealed extensive ulcerative pangastritis with fibrin-covered ulcers. Histopathology confirmed chronic active gastritis with neutrophilic microabscesses and high rates of glandular apoptosis, a hallmark of ICI-induced injury, while excluding infectious etiologies. Following pembrolizumab discontinuation and high-dose corticosteroid therapy, the patient achieved clinical remission, with follow-up endoscopy at eight weeks demonstrating significant mucosal healing. This case emphasizes the need for high clinical suspicion and early endoscopy in patients on immunotherapy with persistent UGI symptoms, as these toxicities often present later than intestinal events and can significantly mimic disease progression.

## Introduction

The therapeutic paradigm for advanced malignancies has been fundamentally redefined by the clinical implementation of immune checkpoint inhibitors (ICIs). These monoclonal antibodies target specific inhibitory signaling pathways, primarily the cytotoxic T-lymphocyte antigen 4 (CTLA-4) and the programmed cell death protein 1 (PD-1)/programmed cell death ligand 1 (PD-L1) axis [1]. By disrupting these signals, ICIs circumvent peripheral tolerance and restore the effector function of exhausted T-cells, facilitating a potent cell-mediated anti-tumor response [1].

Despite their oncological efficacy, the systemic disinhibition of the immune system frequently results in immune-related adverse events (irAEs). The pathophysiology of irAEs is driven by T-cell receptor cross-reactivity between tumor and healthy antigens, leading to autoreactive lymphocytic infiltration and pro-inflammatory cytokine release [1,2]. While gastrointestinal toxicities - predominantly immune-mediated enterocolitis - and hepatobiliary toxicities are common, upper gastrointestinal (UGI) manifestations remain an underreported and distinct clinical entity [2,3].

Isolated immune-related gastritis occurs in an estimated 3% to 5.4% of patients receiving anti-PD-1/PD-L1 therapy [2]. Unlike the early-onset kinetics typical of ICI-induced colitis, UGI toxicities frequently exhibit a delayed presentation, often manifesting months after the initiation of therapy [3,4]. The clinical spectrum ranges from mild dyspepsia to severe ulcerative disease with significant risk of hemorrhage or gastric outlet obstruction. Given that symptoms such as epigastralgia and weight loss frequently overlap with the systemic manifestations of advanced malignancy, a high index of clinical suspicion is requisite [3,5]. This report describes a case of severe ulcerative gastritis following pembrolizumab therapy, illustrating the unique challenges of UGI irAEs and the pivotal role of integrated endoscopic and histopathological assessment in identifying this underrecognized and clinically significant toxicity.

## Case presentation

A 72-year-old female with a 50-pack-year smoking history and metastatic lung adenocarcinoma, with bone and central nervous system involvement, presented with intractable gastrointestinal symptoms. Her oncological treatment included stereotactic radiosurgery (SRS) for four brain lesions and maintenance monotherapy with pembrolizumab (200 mg every three weeks).

Eight months after the initiation of pembrolizumab therapy, the patient presented with a four-week history of progressive postprandial severe nausea and vomiting, persistent dyspepsia, and an 8 kg weight loss (11% of total body weight). Physical examination on admission revealed signs of dehydration and localized epigastric tenderness. Laboratory evaluation was significant for a profound inflammatory state, with a C-reactive protein (CRP) of 157.7 mg/L and marked hypoalbuminemia (2.2 g/dL). Additionally, the patient developed iron-deficiency anemia, with hemoglobin levels reaching a nadir of 6.9 g/dL. Due to the inability to maintain oral intake and the clinical severity, the patient was hospitalized for intravenous hydration and the placement of a nasoenteric tube for nutritional support.

**Figure 1:**
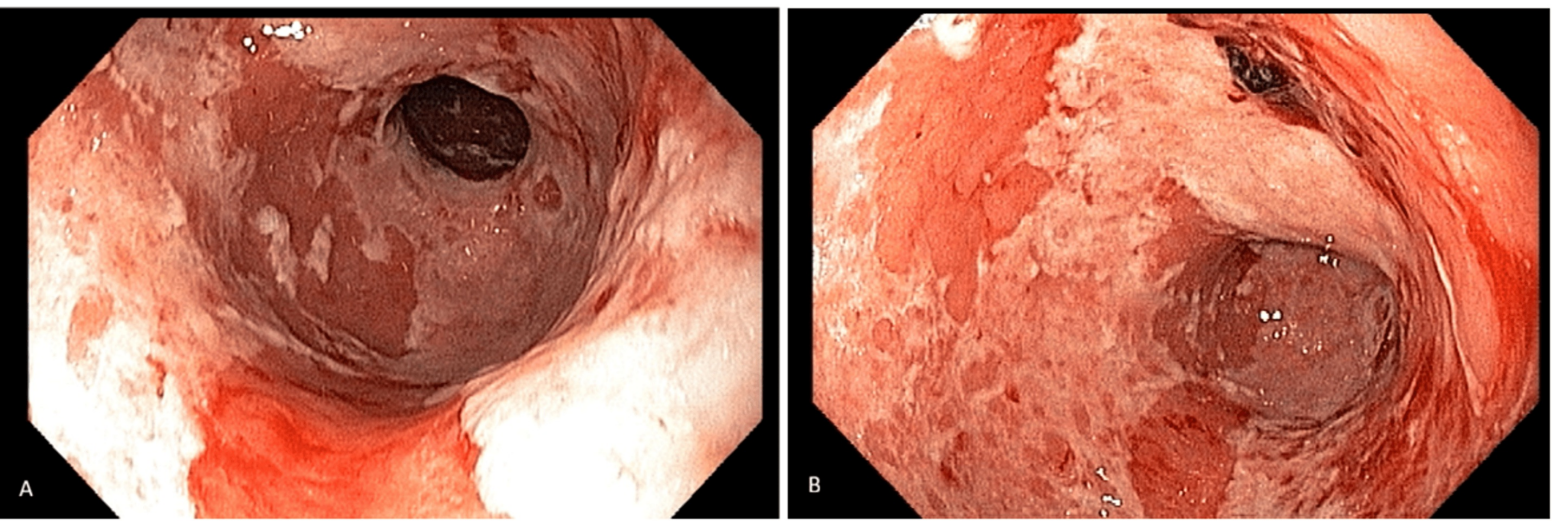
Endoscopic findings of severe immune-related gastritis at initial presentation. (A) View of the gastric antrum and pylorus showing diffuse mucosal edema, marked friability, and extensive geographic ulcerations covered by thick fibrin exudates. (B) Distal gastric body demonstrating similar patterns of severe ulcerative inflammation and mucosal erythema.

To investigate the persistent UGI symptoms, an esophagogastroduodenoscopy (EGD) was performed. The procedure revealed a severe, diffuse ulcerative pangastritis and mild erosive bulboduodenitis. Macroscopic findings included marked mucosal friability, diffuse edema, and multiple geographic superficial ulcers covered by thick fibrin exudates. These findings were consistent with a high-grade irAE (Figure 1).

Gastric body and antrum biopsies were performed to differentiate the etiology of the ulcerative process. Histopathological examination revealed a pattern of active chronic gastritis characterized by a dense lymphoplasmacytic infiltration of the lamina propria, associated with neutrophilic microabscesses. A key diagnostic feature was the presence of prominent glandular apoptosis and intraepithelial lymphocytosis, which are recognized histological hallmarks of immune-mediated mucosal injury. Special stains and immunohistochemistry for *Helicobacter pylori*, cytomegalovirus (CMV), and herpes simplex virus (HSV) were negative, effectively ruling out opportunistic infections and direct neoplastic infiltration.

Following the confirmation of high-grade immune-related gastritis, pembrolizumab was permanently discontinued. The patient was started on high-dose systemic corticosteroids (prednisone 1 mg/kg/day) and intensive acid suppression with double-dose proton pump inhibitors (PPIs). Significant clinical improvement was observed within the first two weeks of therapy, marked by the resolution of vomiting, weight stabilization, and the successful transition from enteral to oral nutrition.

**Figure 2:**
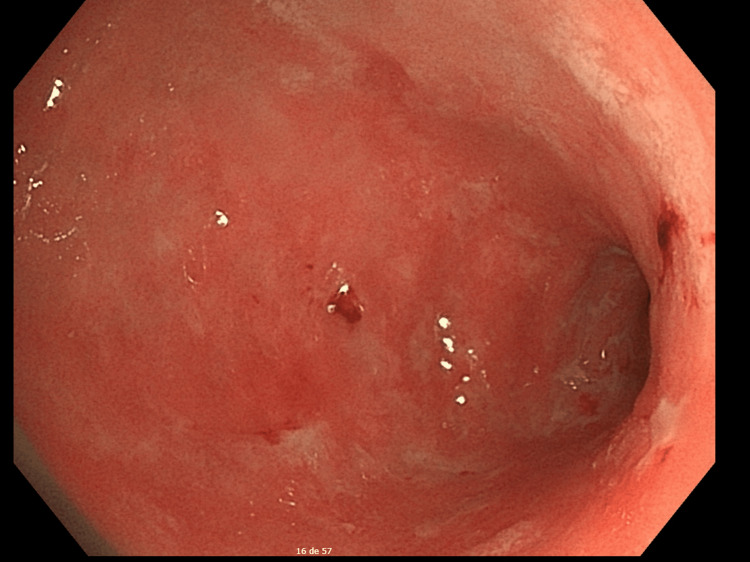
Follow-up endoscopy after eight weeks of systemic corticosteroid therapy, demonstrating expressive mucosal improvement and significant re-epithelialization of the previously ulcerated areas.

A follow-up endoscopy was performed eight weeks after the initiation of immunosuppressive therapy to evaluate mucosal recovery. The repeat EGD demonstrated expressive endoscopic improvement compared to the initial examination, showing a significant reduction in the size and depth of the ulcerative lesions, with widespread signs of mucosal re-epithelialization and a marked decrease in inflammatory activity throughout the gastric segments (Figure 2).

Following documented mucosal healing, the systemic corticosteroid dose was tapered over six weeks. Long-term follow-up confirmed durable resolution of gastrointestinal symptoms and full nutritional recovery. The patient was thereafter transitioned to subsequent lines of antineoplastic treatment, maintaining a stable clinical status with no evidence of relapsing immune-mediated toxicity.

## Discussion

The clinical implementation of ICIs has revolutionized the treatment of advanced malignancies. However, their use is accompanied by a distinct spectrum of immune-related toxicities, stemming from the loss of peripheral self-tolerance [1]. While the gastrointestinal tract is a primary site for such toxicities, significant clinico-pathological heterogeneity exists between the upper and lower segments. Unlike immune-mediated colitis, which is relatively common, isolated severe ulcerative gastritis represents a rare clinical phenotype, with an incidence rate estimated between 3.0% and 5.4% in major retrospective cohorts [2-5].

The pathophysiology of pembrolizumab-induced gastritis is rooted in the disruption of the PD-1/PD-L1 signaling axis, which is constitutively expressed in the gastric mucosa to modulate local immune homeostasis [6]. The pharmacological blockade of this pathway facilitates the expansion of autoreactive CD8+ T-cell clones and triggers a pro-inflammatory cytokine cascade, primarily driven by interferon-gamma (IFN-γ) and tumor necrosis factor-alpha (TNF-α). This process results in direct cytotoxic damage to the epithelium, manifesting as the extensive, geographic ulcerations observed in this case [1,7].

A critical feature of this case is the protracted latency of the onset. While most irAEs follow an early kinetic profile, UGI toxicities often demonstrate a delayed presentation. In our patient, the emergence of severe symptoms eight months post-initiation aligns with the reported median onset of four to nine months for isolated gastric injury [4,7]. This temporal lag poses a significant diagnostic challenge, as symptoms such as dyspepsia and weight loss are frequently misinterpreted as disease progression or paraneoplastic phenomena, potentially delaying the administration of life-saving immunosuppression [4,6].

Diagnostic precision in immune-mediated gastritis necessitates a multidisciplinary approach. Endoscopically, high-grade injury is characterized by diffuse pangastritis with marked mucosal friability and fibrin-covered ulcers. However, histopathology remains the gold standard for diagnostic confirmation. The identification of histological hallmarks-dense lymphoplasmacytic infiltration of the lamina propria, neutrophilic microabscesses, and, most crucially, prominent glandular apoptosis-differentiates this entity from conventional peptic disease or malignancy [3,7,8].

Furthermore, the rigorous exclusion of opportunistic pathogens, particularly CMV and *Helicobacter pylori*, via immunohistochemistry is an absolute prerequisite before escalating to systemic immunosuppressive regimens [5,8].

The therapeutic algorithm for severe irAEs centers on the rapid attenuation of the T-cell-mediated inflammatory microenvironment. Clinical guidelines advocate for the permanent cessation of the offending ICI and the immediate initiation of high-dose systemic corticosteroids (1-2 mg/kg/day of prednisone or equivalent) [5,6]. The rapid clinical and endoscopic resolution documented in this patient underscores the high degree of steroid sensitivity - approximately 80% - observed in isolated ICI-induced gastritis [4,5,8].

The adjunctive use of intensive acid suppression via double-dose PPIs is essential, not as a primary treatment for the immune response, but as a facilitator of mucosal re-epithelialization and a prophylactic measure against steroid-induced peptic ulceration [3,5,8]. Moreover, the implementation of a structured, six-week corticosteroid taper is vital to prevent "rebound" inflammation, which often presents with heightened clinical severity [5].

From an oncological perspective, although the permanent discontinuation of pembrolizumab is mandatory for severe toxicities, the development of severe irAEs has been increasingly recognized as a potential surrogate marker for robust anti-tumor immunity and favorable long-term survival [1,2,4]. The successful management of this serious gastrointestinal complication allowed for the stabilization of the patient's nutritional and performance status, facilitating the transition to subsequent antineoplastic lines while mitigating the risks of hemorrhage or perforation [4,7].

## Conclusions

Severe isolated immune-mediated gastritis is a rare but clinically significant toxicity of pembrolizumab, characterized by a protracted latency period that necessitates high clinical suspicion regardless of treatment duration. Prompt endoscopic evaluation and histopathological confirmation are essential to differentiate immune-related injury from opportunistic infections or neoplastic progression, ensuring the timely initiation of immunosuppression. As demonstrated, early intervention with high-dose corticosteroids and intensive acid suppression can achieve expressive mucosal healing and clinical stabilization. This proactive approach is vital to mitigate serious gastrointestinal complications, allowing for a safe transition to subsequent lines of antineoplastic treatment while preserving the patient’s performance status.
